# Supplementary material for: MetaRibo-Seq measures translation in microbiomes
Source: Nat Commun. 2020 Jun 29;11:3268. doi: 10.1038/s41467-020-17081-z (PMC7324362; doi:10.1038/s41467-020-17081-z)
Supplement: Supplementary file 10 — Supplementary Data 7 [file 41467_2020_17081_MOESM10_ESM.zip › File2/Confidence_High_Taxonomy/277389_out.krona.html]

Javascript must be enabled to view this page.

members
magnitude
magnitudeUnassigned
count
unassigned
taxon
rank

277389\_out

11

2
superkingdom
11

1239
phylum
11

186801
class
11

186802
order
1
11

SRS015431\_contig\_number\_67855

541000
family
2

1263
genus
2

1816688

SRS020869\_contig\_number\_contig-100\_4115.187013SRS148721\_contig\_number\_30561
2
species

186803
family
8

genus
8
572511

765821
species

SRS013965\_contig\_number\_29904SRS014287\_contig\_number\_13368SRS015217\_contig\_number\_28615SRS015578\_contig\_number\_15244SRS017521\_contig\_number\_3033SRS018427\_contig\_number\_14609SRS019068\_contig\_number\_2998SRS146813\_contig\_number\_28946
8
